# Predictors for regression and progression of actively surveilled cervical intraepithelial neoplasia grade 2: A prospective cohort study

**DOI:** 10.1111/aogs.15032

**Published:** 2025-02-10

**Authors:** Laura Bergqvist, Anni Virtanen, Ilkka Kalliala, Ralf Bützow, Maija Jakobsson, Annu Heinonen, Karolina Louvanto, Joakim Dillner, Pekka Nieminen, Karoliina Aro

**Affiliations:** ^1^ Department of Obstetrics and Gynecology University of Helsinki and Helsinki University Hospital Helsinki Finland; ^2^ Department of Pathology University of Helsinki and HUS Diagnostic Center, Helsinki University Hospital Helsinki Finland; ^3^ Department of Metabolism, Digestion and Reproduction, Faculty of Medicine Institute of Reproductive and Developmental Biology, Imperial College London UK; ^4^ Department of Obstetrics and Gynecology Hyvinkää Hospital, Helsinki University Hospital and University of Helsinki Hyvinkää Finland; ^5^ Department of Obstetrics and Gynecology Tampere University Hospital Tampere Finland; ^6^ Department of Obstetrics and Gynecology, Faculty of Medicine and Health Technology Tampere University Tampere Finland; ^7^ Department of Laboratory Medicine Karolinska Institute Stockholm Sweden

**Keywords:** active surveillance, cervical cytology, cervical intraepithelial neoplasia grade 2 (CIN2), colposcopy, HPV genotype, large loop excision of the transformation zone (LLETZ)

## Abstract

**Introduction:**

To evaluate predicting clinical factors for regression and progression of cervical intraepithelial neoplasia (CIN) grade 2 (CIN2) in young women during two years of active surveillance.

**Material and Methods:**

This was a single‐center prospective observational cohort study. Women under 31 years of age giving written informed consent with histologically confirmed CIN2 were followed with colposcopy, cytology, and biopsies every 6 months up to 24 months. At baseline, HPV genotyping was performed on cervical samples. The rates of regression and progression were recorded for every timepoint and at the end of study overall and stratified according to clinical factors and HPV genotypes at baseline. Risk ratio (RR) was used to estimate the relative risks for regression and progression. The study was registered in the ISRCTN registry (ISRCTN91953024).

**Results:**

In total, 205/243 (84.4%) women completed the study. Complete regression (normal histology and/or normal or atypical squamous cells of undetermined significance (ASC‐US) cytology) was detected in 64.4.% (*n* = 132) while 16.1% (*n* = 33) of the lesions progressed to CIN grade 3 (CIN3) or worse including 31 CIN3 cases, one adenocarcinoma in situ and one cervical cancer case. Factors associated with progression were initial large (>50% of the transformation zone) lesion size, risk ratio (RR) 3.06 (95% confidence interval (CI) 1.40–6.69), and high‐grade referral cytology RR 4.73 (95% CI 1.18–19.03). Compared with baseline HPV negativity or having only low‐risk HPV genotypes present, high‐risk HPV (hrHPV) positivity was associated with lower likelihood of regression RR 0.74 (95% CI 0.60–0.91). Age, cigarette smoking, use of combined oral contraceptives or baseline high‐risk HPV genotype, including HPV16, were not associated with the outcomes.

**Conclusions:**

The majority of CIN2 lesions regress in young women. Women with large lesions and/or high‐grade referral cytology should perhaps more often be treated instead of active surveillance. Initial hrHPV genotype does not appear to predict outcomes while not harboring hrHPV favors regression.

## Graphical abstract

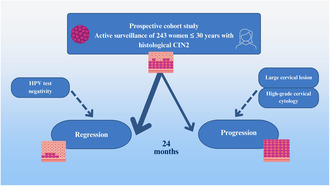

**Abbreviations**
ASC‐ H: atypical squamous cells; cannot exclude high‐grade squamous intraepithelial lesion
ASC‐US: atypical squamous cells of undetermined significance
CIN1: cervical intraepithelial neoplasia grade 1
CIN2: cervical intraepithelial neoplasia grade 2
CIN3: cervical intraepithelial neoplasia grade 3
HPV: Human papilloma virus
Hr: high risk
HSIL: high‐grade squamous intraepithelial lesion
LLETZ: large loop excision of the transformation zone
LSIL: low‐grade squamous intraepithelial lesion
RR: risk ratio

**Key message**

Over 60% of cervical intraepithelial neoplasia grade 2 lesions regress in young women. Lesion size and initial cytology can predict progression of cervical intraepithelial neoplasia grade 2. High‐risk HPV negativity at baseline increases the likelihood of regression.

## 1 INTRODUCTION

Active surveillance of cervical HSIL/CIN2 (high‐grade squamous intraepithelial lesion/cervical intraepithelial neoplasia (CIN) grade 2 (CIN2)) in women of reproductive age has been adapted to several clinical guidelines.^1^, ^2^, ^3^ In general, HSIL lesions, comprising CIN2 and CIN grade 3 (CIN3), are treated with local excision to prevent cancer.^1^, ^2^, ^3^ In young women, however, spontaneous regression of CIN2 has been reported to be up to 50–60% and progression to invasion appears to be rare.^4^, ^5^, ^6^, ^7^ Also, local excisional procedures have been associated with reproductive complications such as increased risk of preterm birth.^8^, ^9^ Robust evidence on clinical parameters for choosing suitable candidates for active surveillance of CIN2 are still missing, but most guidelines suggest restricting this approach to young women and to a maximum period of two years.^1^, ^2^, ^3^

There are several caveats in active surveillance: potential lack of representativeness of biopsies and the subjective nature of histopathological diagnosis^10^, ^11^, ^12^ can lead to untreated CIN3 lesions with substantial potential for progression to invasion.^13^ Costs and demands on the healthcare system may also increase when applying active surveillance protocols as discharge to routine screening even after initial regression of CIN2 can be delayed due to persisting low‐grade lesions or human papillomavirus (HPV) infection.^7^

Overall, the key clinical issue in CIN2 management is identifying more reliably which women should be treated, and which can safely be monitored. The main objective of this study was to examine in a prospective setting the regression and progression rates of CIN2 during active surveillance and factors associated with the outcomes.

## 2 MATERIAL AND METHODS

Women 30 years or younger with histologically confirmed HSIL/CIN2 were asked to participate in a prospective cohort study of active surveillance at Helsinki University Hospital between September 2013 and September 2018. Written informed consent was provided by all participants and large loop excision of the transformation zone (LLETZ) was omitted during the recruitment visit. The study is registered in the ISRCTN registry ISRCTN91953024.

Additional inclusion criteria were transformation zone type 1 or 2, i.e. a visible squamocolumnar junction, and lesion size maximum of three quadrants (75%) of the cervix. Exclusion criteria were pregnancy or lactation at the time of enrolment, human immunodeficiency virus positivity, immunosuppressive medication, previous cervical cancer or CIN3, and concomitant high‐grade vaginal or vulvar disease. The final decision of active surveillance and evaluation of study inclusion and exclusion criteria was at the discretion of the senior colposcopist. The national school‐based HPV vaccination program was not implemented for birth cohorts participating in this study. During the study period, organized screening in the greater Helsinki area began at the age of 25, younger women referred to colposcopy had opportunistic screening at healthcare centers or the private sector.

Women were followed up with colposcopy every 6 months (± 90 days of scheduled visit accepted) up to 24 months (−90 to +120 days accepted). In cases of disease regression prior to 24 months, women were invited to attend the full 24 months of follow‐up and in cases treated for progression further follow‐up visits within the study were omitted. All colposcopies were conducted or overseen by experienced senior colposcopists. Cervical cytology, biopsies, and endocervical curettage were taken at the discretion of the colposcopist. At enrolment, a cervical brush sample for HPV genotyping was obtained. The visit at 18 months could be omitted in women with normal histology and cytology at 12 months. Progression to HSIL/CIN3 or worse (CIN3+; CIN3, adenocarcinoma in situ, squamous cell carcinoma and adenocarcinoma) led to LLETZ treatment. Persistent HSIL/CIN2 and often also CIN grade 1 (CIN1) at the end of the two‐year period was treated with LLETZ. If a woman wished to discontinue surveillance, LLETZ was performed. All histological and cytological samples were processed and analyzed as part of the routine diagnostic process in a university hospital pathology laboratory and reviewed by pathologist specialized in gynecological pathology. At the request of the treating colposcopist, the histological diagnosis was confirmed by an expert gynecological pathologist or evaluated in a multidisciplinary team meeting before the decision of active surveillance. Colposcopists were unaware of HPV genotyping results as these were not available until after the study period.

Information on referral cytology, relevant medical background, clinical findings and procedures, and histopathological results were collected from the institution's electronic medical records. Cervical cytology results were classified according to the Bethesda system. Colposcopic impression was detailed with Reid's colposcopic index at that time. In histological results, neoplastic epithelial lesions were reported as CIN1, CIN2, and CIN3 or low‐grade squamous intraepithelial lesion LSIL/CIN1, HSIL/CIN2, HSIL/CIN3, adenocarcinoma in situ, squamous cell carcinoma and adenocarcinoma. Lesions that were originally given a diagnosis not in accordance with the WHO 2014 tumor classification^14^ were re‐evaluated by the consensus of two expert gynecological pathologists. Non‐neoplastic (eg, reactive, inflammatory) findings and biopsies with no histopathological findings were grouped as “normal histology”. Cytological criteria for colposcopy referral and timing of colposcopy are described in Appendix S1.

Genotyping tested for 14 high‐risk and 20 low‐risk HPV genotypes. The cells collected with a brush from the endocervix were stored at −20°C in sample transport medium (STM; Qiagen GMBH, Germany). The samples were later divided into three aliquots and stored at −80°C. HPV genotyping was done at the Karolinska Institute, Stockholm, Sweden, with the Luminex assay as previously described.^15^

The main outcome measure was the overall regression rate to normal at 24 months and secondary outcome measures histological progression to HSIL/CIN3 or worse and regression to LSIL or normal at 24 months. We also examined factors associated with the outcomes (regression, progression) and the number of LLETZ procedures performed for any reason, and the number of losses to follow‐up. Regression was defined as normal histology on biopsy and normal or atypical squamous cells of undetermined significance (ASC‐US) on cytology. Partial regression was defined as histological LSIL and/or cytological LSIL. Histologically confirmed HSIL/CIN3 or worse at any visit was classified as progression. Persistence was defined as HSIL/CIN2 and/or cytological atypical squamous cells, cannot exclude HSIL (ASC‐H) or HSIL. High‐grade cytology despite low‐grade histology, for example, CIN1 with ASC‐H cytology, was classified as persistence. Rates of regression, persistence, and progression were evaluated at every 6 months (6, 12, 18, 24 months).

High‐risk HPV (hrHPV) positivity was defined as a finding of HPV16, 18, 31, 33, 35, 39, 45, 51, 52, 56, 58, 59, 66, and/or 68 on genotyping. Low‐risk HPV (lrHPV) positivity was defined as a finding of only other HPV's than high‐risk genotypes (Appendix S1). A partial hierarchical system was used to evaluate the prevalence of single HPV genotypes when multiple HPV genotypes were present. All women with HPV16 detected were categorized as HPV16 positive (HPV16+) regardless of other possible genotypes. Cases categorized as HPV18+, 31+, 33+, 45+, 52+ etc. did not have HPV16 present, but could have other high‐ or low‐risk genotypes present. Other hrHPV positivity was defined as other high‐risk genotypes (HPV18, 31, 33, 35, 39, 45, 51, 52, 56, 58, 59, 66, and 68) present than HPV16 regardless of presence of low‐risk genotypes (Table S1).

### 2.1 Statistical analyses

Baseline characteristics and findings of women completing and defaulting the follow‐up were compared using the Chi‐square test or Fisher's exact test, as appropriate. Differences in the frequency of progression and regression between different HPV groups, single genotypes and other factors were compared with risk difference and risk ratio (RR). All statistical tests were two‐sided, with p‐values <0.05 considered statistically significant. All analyses were performed using STATA/SE 15 (StataCorp, College Station, TX, USA).

## 3 RESULTS

**FIGURE 1:**
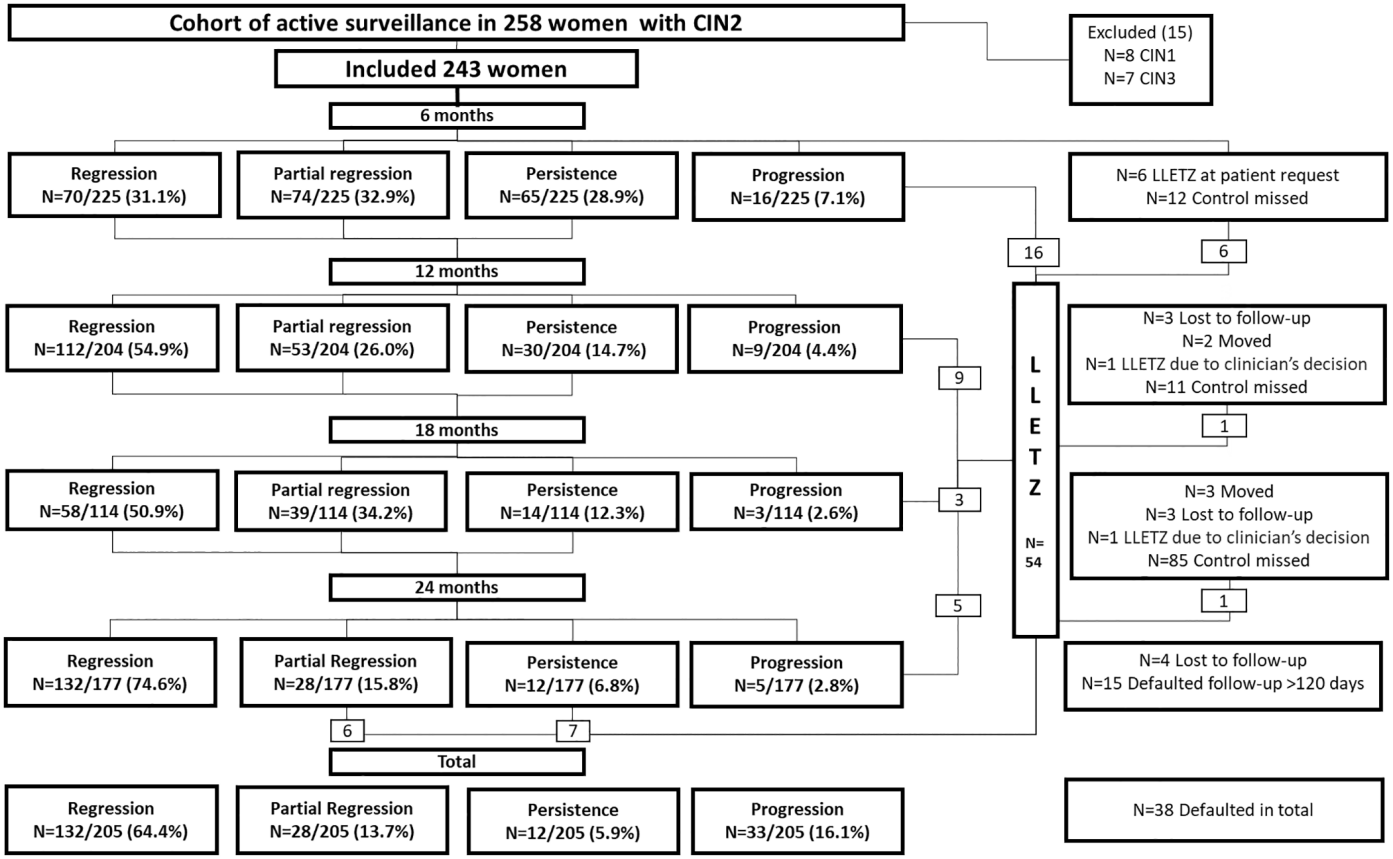
Flow‐chart on the progress and outcomes of prospective cohort study on active surveillance of CIN2. Regression defined as having normal histology and/or cytology negative for intraepithelial lesion or malignancy (NILM) or atypical squamous cells of undetermined significance (ASC‐US). Partial regression defined as having histological cervical intraepithelial neoplasia (CIN) grade 1 (CIN1) and/or cytology low grade squamous intraepithelial lesion (LSIL). Persistence defined as having histological CIN2 and/or atypical squamous cells, cannot exclude high grade squamous intraepithelial lesion (HSIL) or HSIL cytology. Progression defined as having histology CIN grade 3 or worse (CIN3+; CIN3, adenocarcinoma in situ, squamous cell carcinoma, adenocarcinoma). CIN2, cervical intraepithelial neoplasia grade 2; LLETZ, large loop excision of the transformation zone.

A total of 258 women with HSIL/CIN2 were initially recruited in the study. Of these, 15 were excluded due to altered diagnosis to LSIL/CIN1 or HSIL/CIN3 after histopathological re‐review (Figure 1). Outcomes for 205 women (84.4%) were available for final analyses as 38 participants defaulted the study. The largest proportion of the defaulted, 15 out of 38 (39.5%), missed the 24‐month visit, attending later than +120 days. Ten women (overall 4.1%, 10/243) did not return for follow‐up visits despite recall(s) and were considered lost to follow‐up (Figure 1).

The baseline characteristics and findings of the 243 women are presented in Table 1 and Table S1–S3. The median age of the recruited women was 26.5 years (range 17.7–30.9) and 224/243 (92.2%) had an HPV genotyping result at baseline available. The majority of women were referred to colposcopy for high‐grade cytology (74.1%, *n* = 180/243), nearly half smoked cigarettes (45.2%, *n* = 109/241), and were using combined oral contraceptives (42.7%, *n* = 103/241). Overall, 82.6% (*n* = 185/224) were hrHPV positive. HPV16 was the most common detected genotype (42.0%, *n* = 94/224). Apart from multiparous women defaulting more often, we found no statistically significant differences in baseline characteristics between women completing or defaulting the study (Table S2).

**TABLE 1:** Baseline characteristics and findings of 243 women recruited in a study of 2‐year active surveillance of cervical intraepithelial neoplasia 2.

	*N* (%)		*N* (%)
Age (*N* = 243)		Biopsies at baseline (*N* = 239)	
Median 26.5, range 17.7–30.9		Median 2, mean 2,5, range 1–4	
18–25 years	109 (44.9)	1–2	133 (55.6)
26–30 years	134 (55.1)	>2	106 (44.4)
Contraception (*N* = 241)		ATZ size (*N* = 242)	
None	38 (15.8)	0%–25%	109 (45.0)
Condom	52 (21.6)	26%–50%	84 (34.7)
COC	103 (42.7)	>50%	49 (20.2)
Other^a^	48 (19.9)	RCI score (*N* = 239)	
Parity (*N* = 239)		RCI 0–3	90 (37.7)
0	180 (75.3)	RCI 4–6	149 (62.3)
1	39 (16.3)	HPV at baseline (*N* = 224)^b^	
>1	20 (8.4)	Positive	195 (87.1)
Cigarette smoking (*N* = 241)		Negative	29 (12.9)
Yes	109 (45.2)	hrHPV+^c^	185 (82.6)
No	132 (54.8)	lrHPV+^d^	10 (4.5)
Referral cytology (*N* = 243)		HPV16+	94 (42.0)
Low‐grade cytology^e^	55 (22.6)	other hrHPV+^f^	91 (40.6)
High‐grade cytology^g^	180 (74.1)	HPV18+	10 (4.5)
NILM	1 (0.4)	HPV31+	29 (12.9)
ASCU‐US	18 (7.4)	HPV33+	12 (5.4)
LSIL	36 (14.8)	HPV35+	7 (3.1)
ASC‐H	85 (35.0)	HPV45+	5 (2.2)
HSIL	94 (38.7)	HPV51+	8 (3.6)
AGC‐NOS	1 (0.4)	HPV52+	16 (7.1)
AGC‐FN	1 (0.4)	HPV58+	8 (3.6)
Not available	7 (2.9)	HPV59+	5 (2.2)
TZ type (*N* = 242)		HPV66+	8 (3.6)
1	175 (72.3)	HPV68+	5 (2.2)
2	67 (27.7)		

Abbreviations: AGC‐FN, atypical glandular cells, favor neoplasia; AGC‐NOS, atypical glandular cells not otherwise specified; ASC‐H, atypical squamous cells, cannot exclude high‐grade squamous intraepithelial lesion; ASC‐US, atypical squamous cells of undetermined significance; ATZ, atypical transformation zone; COC, combined oral contraceptives; HPV, human papillomavirus; hr, high risk; HSIL, high‐grade squamous intraepithelial lesion; lr, low risk; LSIL, low‐grade squamous intraepithelial lesion; NILM, negative for intraepithelial lesion or malignancy; RCI, Reid's colposcopic index; TZ, transformation zone.

^a^ Including progestin pills, levonorgestrel‐releasing intrauterine system, progestin implant and copper‐releasing intrauterine system.

^b^ Single genotypes presented with all women positive for HPV16 categorized as HPV16+ irrespective of other genotypes detected, for other single genotypes (i.e. HPV18+) cases positive also for HPV16 excluded, multiple infections with other genotypes allowed.

^c^ hrHPV, high‐risk HPV genotypes: 16, 18, 31, 33, 35, 39, 45, 51, 52, 56, 58, 59, 66, and/or 68.

^d^ lrHPV, low‐risk HPV genotypes: 6, 11, 30, 40, 42, 43, 53, 61,67, 69, 70, 73, 74, 81, 83, 86, 87, 89, 90, and/or 91; cases with presence of hrHPV genotypes excluded.

^e^ Low‐grade cytology including ASC‐US, LSIL, AGC‐NOS.

^f^ Other hrHPV genotypes: 18, 31, 33, 35, 39, 45, 51, 52, 56, 58, 59, 66, and/or 68; i.e. cases with HPV16 excluded.

^g^ High‐grade cytology including ASC‐H, HSIL, AGC‐FN.

At 24 months, 64.4% (*n* = 132/205) of women regressed to normal whereas partial regression (histological LSIL and/or cytological LSIL) was found in 13.7% (*n* = 28/205) of women (Table 2). The result of complete regression was based on histology in 92.4% (*n* = 122/132). Women without histological confirmation (*n* = 10) had normal cytology in nine cases and one case of ASC‐US. In total, 16.1% (*n* = 33/205) of the women progressed to HSIL/CIN3 or worse during the study period, most of which (75.8%, *n* = 25/33) were detected and treated within 12 months of enrolment (Table 2, Figure 1). The majority of progressions were to CIN3 (*n* = 31/33), and in addition one case of adenocarcinoma in situ and one squamous cell carcinoma were detected. A total of 85.9% (176/205) of study population had previous cytology before the one leading to referral, of which 92 were negative for intraepithelial lesion or malignancy, 52 were ASC‐US, 26 were LSIL, four were ASC‐H and two were HSIL. While women with normal previous cytology before the referral cytology had higher rate of progression 23.9% compared with women whose previous cytology before the referral was ASC‐US or LSIL 11.5%, the rates of regression were almost similar between these two groups, 62.0% vs 64.1%, respectively. All women with high‐grade cytology preceding the referral one experienced regression.

**TABLE 2:** Outcomes according to characteristics and baseline findings during 2‐year active surveillance of CIN2.

	Complete regression N (%)	Partial regression N (%)	Persistence N (%)	Progression N (%)	Total N
All	132 (64.4)	28 (13.7)	12 (5.9)	33 (16.1)	205
Age					
18–25	54 (60.7)	17 (19.1)	6 (6.7)	12 (13.5)	89
26–30	78 (67.2)	11 (9.5)	6 (5.2)	21 (18.1)	116
Contraception					
COC use	60 (66.7)	15 (16.7)	2 (2.2)	13 (14.4)	90
No COC use	72 (62.6)	13 (11.3)	10 (8.7)	20 (17.4)	115
Parity					
0	100 (63.7)	26 (16.6)	7 (4.5)	24 (15.3)	157
≥ 1	30 (66.7)	2 (4.4)	5 (11.1)	8 (17.8)	45
Cigarette smoking					
Yes	58 (63.0)	15 (16.3)	8 (8.7)	11 (12.0)	92
No	74 (65.5)	13 (11.5)	4 (3.5)	22 (19.5)	113
Referral cytology					
Low grade^a^	34 (72.3)	7 (14.9)	4 (8.5)	2 (4.3)	47
High grade^b^	95 (61.7)	20 (13.0)	8 (5.2)	31 (20.1)	154
ASC‐US	11 (68.8)	3 (18.8)	1 (6.3)	1 (6.3)	16
LSIL	22 (73.3)	4 (13.3)	3 (10.0)	1 (3.3)	30
ASC‐H	52 (68.4)	10 (13.2)	1 (1.3)	13 (17.1)	76
HSIL	43 (55.8)	10 (13.0)	7 (9.1)	17 (22.1)	77
AGC‐NOS	1 (100.0)	0 (0.0)	0 (0.0)	0 (0.0)	1
AGC‐FN	0 (0.0)	0 (0.0)	0 (0.0)	1 (100.0)	1
TZ type					
1	99 (66.0)	21 (14.0)	9 (6.0)	21 (14.0)	150
2	33 (60.0)	7 (12.7)	3 (5.5)	12 (21.8)	55
ATZ size					
0%–25%	70 (74.5)	11 (11.7)	4 (4.3)	9 (9.6)	94
26%–50%	45 (64.3)	8 (11.4)	5 (7.1)	12 (17.1)	70
>50%	17 (41.5)	9 (22.0)	3 (7.3)	12 (29.3)	41
RCI score					
RCI 0–3	51 (66.2)	12 (15.6)	5 (6.5)	9 (11.7)	77
RCI 4–6	80 (64.0)	15 (12.0)	6 (4.8)	24 (19.2)	125
Baseline biopsies					
1–2	76 (70.4)	14 (13.0)	3 (2.8)	15 (13.9)	108
>2	53 (56.4)	14 (14.9)	9 (9.6)	18 (19.1)	94
HPV at baseline					
Positive	101 (62.3)	23 (14.2)	10 (6.2)	28 (17.3)	162
Negative	20 (76.9)	3 (11.5)	1 (3.8)	2 (7.7)	26
hrHPV+^c^	94 (60.6)	23 (14.8)	10 (6.5)	28 (18.1)	155
Other hrHPV+^d^	44 (58.7)	17 (22.7)	4 (5.3)	10 (13.3)	75
HPV16+	50 (62.5)	6 (7.5)	6 (7.5)	18 (22.5)	80
HPV18+	4 (40.0)	5 (50.0)	0 (0.0)	1 (10.0)	10
HPV31+	13 (59.1)	3 (13.6)	1 (4.5)	5 (22.7)	22
HPV33+	2 (25.0)	2 (25.0)	2 (25.0)	2 (25.0)	8
HPV45+	1 (25.0)	2 (50.0)	1 (25.0)	0 (0.0)	4
HPV52+	8 (66.7)	4 (33.3)	0 (0.0)	0 (0.0)	12
lrHPV+^e^	7 (100.0)	0 (0.0)	0 (0.0)	0 (0.0)	7

*Note*: Complete regression defined as having normal histology and/or cytology negative for intraepithelial lesion or malignancy/ASC‐US at end of surveillance. Partial regression defined as having histological cervical intraepithelial neoplasia (CIN) grade 1 and/or cytology LSIL at end of surveillance. Persistence defined as having histological CIN grade 2 and/or ASC‐H/HSIL cytology at end of surveillance. Progression defined as having a finding of CIN grade 3 or worse at any time during surveillance.

Abbreviations: AGC‐FN, atypical glandular cells, favor neoplasia; AGC‐NOS, atypical glandular cells not otherwise specified; ASC‐H, atypical squamous cells, cannot exclude high‐grade squamous intraepithelial lesion; ASC‐US, atypical squamous cells of undetermined significance; ATZ, atypical transformation zone; CIN2, Cervical intraepithelial neoplasia grade 2; COC, combined oral contraceptives; HPV, human papillomavirus; HSIL, high‐grade squamous intraepithelial lesion; LSIL, low‐grade squamous intraepithelial lesion; RCI, Reid's colposcopic index; TZ, transformation zone.

^a^ Low‐grade cytology including ASC‐US, LSIL, AGC‐NOS.

^b^ High‐grade cytology including ASC‐H, HSIL, AGC‐FN.

^c^ hrHPV, high‐risk HPV genotypes: 16, 18, 31, 33, 35, 39, 45, 51, 52, 56, 58, 59, 66, and/or 68.

^d^ Other hrHPV genotypes: 18, 31, 33, 35, 39, 45, 51, 52, 56, 58, 59, 66, and/or 68; i.e. cases with HPV16 excluded.

^e^ lrHPV, low‐risk HPV genotypes: 6, 11, 30, 40, 42, 43, 53, 61,67, 69, 70, 73, 74, 81, 83, 86, 87, 89, 90, and/or 91; cases with presence of hrHPV genotypes excluded.

Relative differences and risk ratios for regression and progression are presented in Table 3 and Table S4. The majority of parameters examined such as age and cigarette smoking did not affect regression or progression. HrHPV‐positive women had lower likelihood of regression RR 0.74 (95% confidence interval (CI) 0.60–0.91, *p* = 0.021) when compared to HPV negativity or presence of only low‐risk HPV genotypes at baseline. Women with more than two cervical biopsies at initial diagnostic colposcopy were less likely to regress RR 0.80 (95% CI 0.65–0.99, *p* = 0.039) than those with fewer biopsies. A large initial cervical lesion (>50%) was associated with lower regression rate, RR 0.56 (95% CI 0.38–0.82, *p* < 0.001) and higher progression rate, RR 3.06 (95% CI 1.40–6.69, *p* = 0.004) when compared to lesions covering ≤25% of the transformation zone. High‐grade referral cytology was also associated with a higher progression rate, RR 4.73 (95% CI 1.18–19.03, *p* = 0.010). HPV16 positivity was associated with 9% higher absolute, although not statistically significant, risk of progression when compared to positivity for other high‐risk genotypes (RR 1.69, 95% CI 0.83–3.42, *p* = 0.138).

**TABLE 3:** Risk differences and risk ratios for regression and progression of characteristics and baseline findings among 205 women with active surveillance of cervical intraepithelial neoplasia 2.

Regression				Progression			
	Risk difference	Risk ratio	*p*		Risk difference	Risk ratio	*p*
	(95% CI)	(95% CI)			(95% CI)	(95% CI)	
*Age*							
18–25 years	Ref	Ref		18–25 years	Ref	Ref	
*N* = 54/89				*N* = 12/89			
26–30 years	0.07	1.11	0.330	26–30 years	0.05	1.34	0.372
*N* = 78/116	(−0.07 to 0.20)	(0.90 to 1.37)		*N* = 21/116	(−0.05 to 0.15)	(0.70 to 2.58)	
*Contraception*							
No COC use	Ref	Ref		No COC use	Ref	Ref	
*N* = 72/115				*N* = 20/115			
COC use	0.04	1.06	0.547	COC use	−0.03	0.83	0.569
*N* = 60/90	(−0.09 to 0.17)	(0.87 to 1.30)		*N* = 13/90	(−0.13 to 0.07)	(0.44 to 1.58)	
*Parity*							
0	Ref	Ref		0	Ref	Ref	
*N*= 100/157				*N* = 24/157			
≥1	0.03	1.05	0.714	≥1	0.02	1.16	0.687
*N* = 30/45	(−0.13 to 0.19)	(0.83 to 1.33)		*N* = 8/45	(−0.10 to 0.15)	(0.56 to 2.41)	
*Cigarette smoking*							
No	Ref	Ref		No	Ref	Ref	
*N* = 74/113				*N* = 22/113			
Yes	−0.02	0.96	0.716	Yes	−0.08	0.61	0.146
*N* = 58/92	(−0.16 to 0.11)	(0.78 to 1.18)		*N* = 11/92	(−0.17 to 0.02)	(0.31 to 1.20)	
*Referral cytology*							
Low grade^a^	Ref	Ref		Low grade^a^	Ref	Ref	
*N* = 34/47				*N* = 2/47			
High grade^b^	−0.11	0.85	0.183	High grade^b^	0.16	4.73	0.010
*N* = 95/154	(−0.26 to 0.04)	(0.69 to 1.06)		*N* = 31/154	(0.07 to 0.24)	(1.18 to 19.03)	
*ATZ size*							
≤25%	Ref	Ref		≤25%	Ref	Ref	
*N* = 70/94				*N* = 9/94			
>50%	−0.33	0.56	<0.001	>50%	0.20	3.06	0.004
*N* = 17/41	(−0.50 to −0.16)	(0.38 to 0.82)		*N* = 12/41	(0.05 to 0.35)	(1.40 to 6.69)	
*Baseline biopsies*							
1–2	Ref	Ref		1–2	Ref	Ref	
*N* = 76/108				*N* = 15/108			
>2	−0.14	0.80	0.039	>2	0.05	1.38	0.313
*N* = 53/94	(−0.27 to −0.01)	(0.65 to 0.99)		*N* = 18/94	(−0.05 to 0.16)	(0.74 to 2.58)	
*HPV at baseline*							
Non‐hrHPV^c^	Ref	Ref		Non‐hrHPV^c^	Ref	Ref	
*N* = 27/33				*N* = 2/33			
hrHPV^d^	−0.21	0.74	0.021	hrHPV^d^	0.12	2.98	0.087
*N* = 94/155	(−0.36 to −0.06)	(0.60 to 0.91)		*N* = 28/155	(0.02 to 0.22)	(0.75 to 11.90)	
Other hrHPV^e^	Ref	Ref		Other hrHPV^e^	Ref	Ref	
*N* = 44/75				*N* = 10/75			
HPV16+	0.04	1.07	0.625	HPV16+	0.09	1.69	0.138
*N* = 50/80	(−0.12 to 0.19)	(0.83 to 1.37)		*N* = 18/80	(−0.03 to 0.21)	(0.83 to 3.42)	
Non‐HPV18/31/33‐ hrHPV^f^	Ref	Ref		Non‐HPV18/31/33 hrHPV^f^	Ref	Ref	
*N* = 71/109				*N* = 18/109			
HPV18/31/33+^g^	−0.16	0.75	0.071	HPV18/31/33+^g^	0.04	1.24	0.573
*N* = 19/39	(−0.34 to 0.02)	(0.53 to 1.06)		*N* = 8/39	(−0.10 to 0.18)	(0.59 to2.63)	

Abbreviations: ATZ: atypical transformation zone; COC: combined oral contraceptives; HPV: human papillomavirus; hr.: high risk.

^a^ Low‐grade cytology including atypical squamous cells of undetermined significance; low‐grade squamous intraepithelial lesion and atypical glandular cell not otherwise specified.

^b^ High‐grade cytology including atypical squamous cells, cannot exclude high‐grade squamous intraepithelial lesion; high‐grade squamous intraepithelial lesions and atypical glandular cells, favor neoplasia.

^c^ Non‐hrHPV including women with only low‐risk HPV or HPV negative women.

^d^ hrHPV including HPV16, 18, 31, 33, 35, 39, 45, 51, 52, 56, 58, 59, 66, and/or 68.

^e^ Other hrHPV including 18, 31, 33, 35, 39, 45, 51, 52, 56, 58, 59, 66, and/or 68; i.e. coinfections with HPV16 excluded.

^f^ Non‐HPV18/31/33 hrHPV including HPV16, 35,39,45,51,52,56,58,59,66 and/or 68.

^g^ Including HPV18, 31 and/or 33; coinfections with HPV16 excluded.

Combinations of clinical factors associated with progression yielded even higher risk ratios than these examined separately (Table S5). HPV16 positivity increased the risk of progression in combination either with large lesion or high‐grade referral cytology compared with HPV16 negativity and small lesion or low‐grade cytology, whereas the combination with positivity for other hrHPV genotypes did not. The risk of progression was the highest with the combination of large lesion and high‐grade referral cytology, RR 7.45 (95% CI 1.79–31.00, *p* = 0.001).

Altogether 54 LLETZ procedures were performed to 228 women (23.7%) with information available at our institution, resulting in 174 women (76.3%) avoiding LLETZ for newly diagnosed HSIL/CIN2 within two years. Thirty‐three of the LLETZ procedures were for disease progression (61.1%, *n* = 33/54) and 13 for persistence or partial regression at the end of the study period (24.1%, *n* = 13/54) (Figure 1). Six additional LLETZ procedures were done for withdrawal of consent and two based on clinical decision (colposcopic impression, symptom of postcoital bleeding) of the senior colposcopist.

Outside the study protocol, 92 of completely regressed women (69.7%, 92/132) were tested for hrHPV with a routine clinical test (Aptima, Hologic, Marlborough, MA, USA) at 24 months. The hrHPV test result was negative for 82.6% (76/92). However, all women without a routine clinical test result had normal histological findings at the end of the study period.

During data analysis some oversights of the original study protocol were detected. Despite use of immunosuppressive medication, three women participated in the study. Evaluation of transformation zone type and lesion size varied sometimes between the diagnostic colposcopy visit and the recruitment visit, and four women had a type 3 transformation zone and three women had a lesion extending to all four cervical quadrants recorded in the electronic records. These women were included in the study population as the final decision of active surveillance and study participation was at the discretion of the senior colposcopist. Of these 10 women fulfilling the original exclusion criteria, 50% regressed completely and 30% progressed. In addition, 32 women (13.2%) became pregnant during the study period. Some study visits were postponed due to pregnancy or delivery. Of the women with pregnancies 75% regressed and 6.3% progressed.

## 4 DISCUSSION

In this prospective cohort study of active surveillance of HSIL/CIN2 for two years in women under 31 years of age, we found a high complete histological regression rate, 64%, while only 16% of lesions progressed including one invasive carcinoma detected (0.4%). Initially large lesion size and high‐grade referral cytology were associated with higher rate of progression, while women not harboring an hrHPV genotype at enrolment were more likely to regress. HPV16 positivity at baseline did not increase the likelihood of progression when compared to other hrHPV genotypes. Overall, three out of four women followed with active surveillance for two years avoided a LLETZ procedure. Loss to follow‐up was rare (4%).

The strengths of this study include the prospective longitudinal study design with high adherence to follow‐up. The study was carried out in a tertiary high‐volume single referral center reducing selection bias. All colposcopies were performed by or under the direct supervision of senior colposcopists and all histopathologic samples were reviewed by pathologists specialized in gynecologic pathology. Diligent recording of clinical features in the electronic databases minimized missing data. HPV genotyping was performed at an international reference laboratory. Still, there are also some limitations in this study: The information on women who refused or were not asked to participate in the study was unavailable. However, detailed information on background factors was available for this selected population of young women contributing to future women selection. Histopathological re‐review of all biopsies during follow‐up was not performed, thus, reflecting a real‐life clinical setting. There were some deviations from the original study protocol regarding women selection, but this did not impact the overall results.

Our finding on the regression^16^, ^17^, ^18^, ^19^ and progression^4^, ^6^, ^17^, ^18^ rates of HSIL/CIN2 are in general consistent with most previous reports, although the regression rate was slightly higher^4^, ^5^, ^6^ and progression rate lower^5^, ^16^ than in some studies. This could be explained by differences in study design and inclusion and exclusion criteria applied. Rate of invasive disease, 0.4%, is also comparable to that of previous reports^4^, ^5^, ^6^, ^7^ and as a rare immediate outcome can be considered acceptable as FIGO stage IA disease can mostly be treated locally sparing fertility with low risk of lymph node metastasis or relapse.^20^

The association of large lesion size with risk of progression could easily be incorporated into clinical practice, as well as the initial cytology. The finding of high‐grade cytology as a predictor of progression or non‐regression is in accord with other recent studies.^5^, ^17^, ^21^, ^22^, ^23^ A relationship between lesion size and progression has not conclusively been showed earlier, but one study has found small lesion size to predict regression of HSIL.^21^, ^24^, ^25^ We found no difference in lesion size between women positive for HPV16 or positive for other hrHPV types (data not shown). Lesions associated with HPV16 have been found to grow more rapidly than those associated with other HPV genotypes.^26^ The observed association of a greater number of biopsies taken at diagnosis with lower regression rate is most likely linked to lesion size as well. We found no statistically significant difference in risk of progression or regression between smokers and non‐smokers despite nearly half of the women being active cigarette smokers in our study population. Our results are in contrast to some previous reports,^21^, ^27^ but not all.^17^, ^28^ It has been demonstrated that smoking is a risk factor for development of cervical precancerous lesions in women with persistent HPV infection.^29^

In contrast to earlier findings, we did not find HPV16 to be robustly associated with the risk of progression when compared to other high‐risk HPV genotypes,^16^, ^17^, ^21^, ^22^ this can partially be attributed to the difference in choice of the comparative group (all those negative for HPV16 vs. those positive for other high‐risk genotypes). Nevertheless, when HPV‐negative women and women only positive for lrHPV's in genotyping were included in the reference group, our result was similar to that of other studies with regard to risk of progression (*p* = 0.035), but not for regression (*p* = 0.646; data not shown). In our study, comparable progression rates to that of HPV16 (23%) were seen for HPV31 (23%) and HPV33 (25%). Also, over 60% of HPV16‐positive women cleared their HSIL/CIN2 lesion completely. However, the results indicate that HPV16 positivity combined with large lesion size or high‐grade referral cytology were associated with higher risk of progression than HPV16 negativity with small lesion size or low‐grade cytology.

HPV16 was the most prevalent genotype detected as expected and the lower detection rates of individual other genotypes can impact the results. Higher sensitivity of genotyping, i.e., lower detection threshold, should be taken to account when interpreting these results. Based on these results, the outcome of a HSIL/CIN2 lesion cannot fully be attributed to infecting HPV genotype. HrHPV negativity, however, could be a good indicator for higher likelihood of regression, which has also been demonstrated in other studies.^6^, ^17^

In a previous study of 2417 women with actively surveilled HSIL/CIN2, over 40% of women required prolonged surveillance rather than discharge to routine screening after 22 months,^7^ which increases costs and demands on the health care system. Based on histology and cytology, 64% (*n* = 132/205) of women with complete clearance of HSIL/CIN2 in this cohort could be considered to have no immediate need for further follow‐up outside routine screening. Due to routine clinical hrHPV testing not being a part of the original study protocol, only approximately two thirds of the women who had complete histological regression had a routine clinical hrHPV test result at the end of the study, with 17% being hrHPV positive and in need of continued short‐interval surveillance. For the untested third of the regressed, hrHPV status and need for continued surveillance based on hrHPV status unfortunately remain unknown.

Implementing an active surveillance protocol most likely requires more colposcopy visits than treatment with LLETZ and recommended test‐of‐cure. Substituting some colposcopy visits of active surveillance of HSIL/CIN2 by cytology and HPV testing only does not seem feasible considering the timing of progression events.

The majority of progressions (76%) were detected early (6–12 months) after enrolment, which has also been a finding in other cohorts.^5^, ^16^ This partly implies that the initial diagnosis of HSIL/CIN2 have been in some cases an underestimation underscoring the clinical difficulty of achieving correct diagnosis. A Danish study found 12% of women initially diagnosed with HSIL/CIN2 being upgraded to HSIL/CIN3 after re‐review.^12^ Losses to follow‐up are therefore a major concern. In this study, with the time points of 6 and 12 months combined, altogether 91.6% (141/154) of those found to have regression (≤CIN1) at 24 months had already regressed and only 12 additional regression cases occurred after that during the study period. These observations raise a question of possibly shortening the recommended active surveillance period of CIN2 from 24 months to 12 months. A recent registry‐based study with 20 years of follow‐up showed a nearly fourfold risk of developing cervical cancer (2.7%) in women with a history of untreated HSIL/CIN2 compared to cases treated immediately (0.8%).^30^ The registry‐based data, however, cannot inform on patient selection and criteria for active surveillance, but does highlight the need for more stringent criteria than those currently applied and the need for long‐term routine screening despite initial regression of HSIL/CIN2 as is the case for those who are treated. For the future, preliminary findings suggest that DNA methylation might be able to discern progressive HSIL/CIN2 lesions from regressive ones, but these findings need further validation before implementation into clinical practice.^25^, ^31^

The finding of increased risk of preterm birth after LLETZ might not fully be associated with the procedure, but the disease itself. Previous studies have found even women with history of colposcopic examination with histological LSIL/CIN1 or less to have increased risk of preterm birth.^9^, ^32^ Consequently, the risk difference of preterm birth between women with and without local treatment of HSIL/CIN2 could be quite small raising questions of the importance of avoiding LLETZ procedures. Taking all into consideration, we probably should aim at active surveillance of HSIL/CIN2 only in women with an 80–90% chance of permanent clearance if reliable criteria for this could be developed.

## 5 CONCLUSION

Regression occurred in majority of CIN2 lesions, in particular in women negative for hrHPV genotypes. Treatment should be considered for women with large cervical lesions and/or high‐grade referral cytology predicting progression. Understanding the contributing factors for regression and progression of cervical lesion has valuable implications on clinical decision‐making of active surveillance.

## AUTHOR CONTRIBUTIONS

Laura Bergqvist: Writing—review and editing, Writing—original draft, Visualization, Methodology, Investigation, Formal analysis, Data curation. Anni Virtanen: Writing—review and editing, Methodology, Investigation, Data curation. Ilkka Kalliala: Writing—review and editing, Validation, Resources, Methodology, Formal analysis. Ralf Bützow: Writing—review and editing, Methodology, Investigation, Data curation. Maija Jakobsson: Writing—review and editing, Methodology, Investigation, Conceptualization. Annu, Heinonen: Writing—review and editing, Investigation, Data curation. Karolina Louvanto: Writing—review and editing, Validation. Joakim Dillner: Writing—review and editing, Validation, Investigation. Pekka Nieminen: Writing—review and editing, Validation, Supervision, Resources, Project administration, Methodology, Conceptualization. Karoliina Aro: Writing—review and editing, Visualization, Methodology, Investigation, Formal analysis, Data curation, Conceptualization.

## FUNDING INFORMATION

This study was funded by the Finnish Medical Society Duodecim (LB), Helsinki Uusimaa Hospital District, Special state funding (IK, PN) and Research Council of Finland (IK).

## CONFLICT OF INTEREST STATEMENT

IK has received a speaker's fee from the association of Finnish private gynecologists outside the work of this study. KA has received a research grant from Astra Zeneca, consulting fees from Gedeon Richter and support for attending a meeting by Johnson & Johnson all outside this study. AH has received Helsinki University research grants outside this study. KL has received research grant from Academy of Finland, Sigrid Juselius Foundation and Finnish Cancer Foundation all outside the work of this study. The remaining authors declare no conflicts of interest.

## ETHICS STATEMENT

The study was approved by Helsinki‐Uusimaa Hospital District Ethical Committee (131/13/03/03/2013;24/4/2013) on April 24, 2013. All women gave written informed consent.

## Supporting information


Appendix S1.

## References

[1] Perkins RB , Guido RS , Castle PE , et al. 2019 ASCCP risk‐based management consensus guidelines for abnormal cervical cancer screening tests and cancer precursors. J Low Genit Tract Dis. 2020;24:102‐131. 32243307 10.1097/LGT.0000000000000525 PMC7147428

[2] Working group set up by the Finnish Medical Society Duodecim and the Finnish Colposcopy Association . Cytological Changes in the Cervix, Vagina and Vulva. Current Care Guidelines. The Finnish Medical Society Duodecim. Accessed October 17, 2023. https://kaypahoito.fi, updated 2023

[3] National Health Service UK . Colposcopic diagnosis, treatment and follow up. Cervical screening: programme and colposcopy management. Accessed October 17, 2023. https://www.gov.uk/government/publications/cervical‐screening‐programme‐and‐col‐poscopy‐management/3‐colposcopic‐diagnosis‐treatment‐and‐follow‐up

[4] Tainio K , Athanasiou A , Tikkinen KAO , et al. Clinical course of untreated cervical intraepithelial neoplasia grade 2 under active surveillance: systematic review and meta‐analysis. BMJ. 2018;360:k499. 29487049 10.1136/bmj.k499 PMC5826010

[5] Lycke KD , Kahlert J , Damgaard RK , et al. Clinical course of cervical intraepithelial neoplasia grade 2: a population‐based cohort study. Am J Obstet Gynecol. 2023;229:656.e1‐656.e15. 10.1016/j.ajog.2023.08.008 37595822

[6] Loopik DL , Bentley HA , Eijgenraam MN , IntHout J , Bekkers RLM , Bentley JR . The natural history of cervical intraepithelial neoplasia grades 1, 2, and 3: a systematic review and meta‐analysis. J Low Genit Tract Dis. 2021;25:221‐231. 34176914 10.1097/LGT.0000000000000604

[7] Silver MI , Gage JC , Schiffman M , et al. Clinical outcomes after conservative Management of Cervical Intraepithelial Neoplasia Grade 2 (CIN2) in women ages 21‐39 years. Cancer Prev Res (Phila). 2018;11:165‐170. 29437696 10.1158/1940-6207.CAPR-17-0293

[8] Kyrgiou M , Athanasiou A , Paraskevaidi M , et al. Adverse obstetric outcomes after local treatment for cervical preinvasive and early invasive disease according to cone depth: systematic review and meta‐analysis. BMJ. 2016;354:i3633. 27469988 10.1136/bmj.i3633 PMC4964801

[9] Athanasiou A , Veroniki AA , Efthimiou O , et al. Comparative effectiveness and risk of preterm birth of local treatments for cervical intraepithelial neoplasia and stage IA1 cervical cancer: a systematic review and network meta‐analysis. Lancet Oncol. 2022;23:1097‐1108. 35835138 10.1016/S1470-2045(22)00334-5 PMC9630146

[10] Dalla Palma P , Giorgi Rossi P , Collina G , et al. The reproducibility of CIN diagnoses among different pathologists: data from histology reviews from a multicenter randomized study. Am J Clin Pathol. 2009;132:125‐132. 19864243 10.1309/AJCPBRK7D1YIUWFP

[11] Stoler MH , Schiffman M . Atypical squamous cells of undetermined significance‐low‐grade squamous intraepithelial lesion triage study (ALTS) group. Interobserver reproducibility of cervical cytologic and histologic interpretations: realistic estimates from the ASCUS‐LSIL triage study. JAMA. 2001;285:1500‐1505. 11255427 10.1001/jama.285.11.1500

[12] Damgaard RK , Jenkins D , Stoler MH , et al. High prevalence of HPV16 and high‐grade cytology in women undergoing active surveillance for cervical intraepithelial neoplasia grade 2. Acta Obstet Gynecol Scand. 2023;102:1227‐1235. 37469102 10.1111/aogs.14627 PMC10407017

[13] McCredie MR , Sharples KJ , Paul C , et al. Natural history of cervical neoplasia and risk of invasive cancer in women with cervical intraepithelial neoplasia 3: a retrospective cohort study. Lancet Oncol. 2008;9:425‐434. 18407790 10.1016/S1470-2045(08)70103-7

[14] Kurman RJ , Carcangiu ML , Herrington CS , Young RH . WHO Classification of Tumours of Female Reproductive Organs. WHO Classification of Tumours. Vol 6. 4th ed. IARCPress; 2014.

[15] Söderlund‐Strand A , Carlson J , Dillner J . Modified general primer PCR system for sensitive detection of multiple types of oncogenic human papillomavirus. J Clin Microbiol. 2009;47:541‐546. 19144817 10.1128/JCM.02007-08 PMC2650955

[16] Kylebäck K , Ekeryd‐Andalen A , Greppe C , Björkenfeldt Havel C , Zhang C , Strander B . Active expectancy as alternative to treatment for cervical intraepithelial neoplasia grade 2 in women aged 25 to 30 years: ExCIN2—a prospective clinical multicenter cohort study. Am J Obstet Gynecol. 2022;227:742.e1‐742.e11. 10.1016/j.ajog.2022.06.051 35777432

[17] Salvadó A , Miralpeix E , Solé‐Sedeno JM , et al. Predictor factors for conservative management of cervical intraepithelial neoplasia grade 2: cytology and HPV genotyping. Gynecol Oncol. 2021;162:569‐574. 34226019 10.1016/j.ygyno.2021.06.019

[18] Loopik DL , Bekkers RLM , Massuger LFAG , Melchers WJG , Siebers AG , Bentley J . Justifying conservative management of CIN2 in women younger than 25 years ‐ a population‐based study. Gynecol Oncol. 2019;152:82‐86. 30413339 10.1016/j.ygyno.2018.10.038

[19] Tjandraprawira KD , Olaitan A , Petrie A , Wilkinson N , Rosenthal AN . Comparison of expectant and excisional/ablative Management of Cervical Intraepithelial Neoplasia Grade 2 (CIN2) in the era of HPV testing. Obstet Gynecol Int. 2022;2022:7955290. 35371262 10.1155/2022/7955290 PMC8970964

[20] Cibula D , Raspollini MR , Planchamp F , et al. ESGO/ESTRO/ESP guidelines for the management of patients with cervical cancer ‐ update 2023. Int J Gynecol Cancer. 2023;33:649‐666. 37127326 10.1136/ijgc-2023-004429 PMC10176411

[21] Sykes PH , Simcock BJ , Innes CR , et al. Predicting regression of cervical intraepithelial neoplasia grade 2 in women under 25 years. Am J Obstet Gynecol. 2022;226:222.e1‐222.e13. 10.1016/j.ajog.2021.09.009 34534506

[22] Damgaard RK , Jenkins D , Stoler MH , et al. Human papillomavirus genotypes and risk of persistence and progression in women undergoing active surveillance for cervical intraepithelial neoplasia grade 2. Am J Obstet Gynecol. 2024;230:655.e1‐655.e10. 10.1016/j.ajog.2024.01.029 38336125

[23] Castle PE , Stoler MH , Solomon D , Schiffmam M . The relationship of community biopsy‐ diagnosed cervical intraepithelial neoplasia grade 2 to the quality control pathology‐reviewed diagnoses: an ALTS report. Am J Clin Pathol. 2007;127:805‐815. 17439841 10.1309/PT3PNC1QL2F4D2VL

[24] Discacciati MG , De Souza CA , D'Otavianno MG , et al. Outcome of expectant management of cervical intraepithelial neoplasia grade 2 in women followed for 12 months. Eur J Obstet Gynecol Reprod Biol. 2011;155:204‐208. 21193261 10.1016/j.ejogrb.2010.12.002

[25] Kremer WW , Dick S , Heideman DAM , et al. Clinical regression of high‐grade cervical intraepithelial neoplasia is associated with absence of FAM19A4/miR124‐2 DNA methylation (CONCERVE study). J Clin Oncol. 2022;40:3037‐3046. 35512257 10.1200/JCO.21.02433 PMC9462536

[26] Wentzensen N , Walker J , Schiffman M , et al. Heterogeneity of high‐grade cervical intraepithelial neoplasia related to HPV16: implications for natural history and management. Int J Cancer. 2013;1(132):148‐154. 10.1002/ijc.27577 PMC3409928 22488167

[27] Loopik DL , Doucette S , Bekkers RL , Bentley JR . Regression and Progression predictors of CIN2 in women younger than 25 years. J Low Genit Tract Dis. 2016;20:213‐217. 27203702 10.1097/LGT.0000000000000215

[28] Moscicki AB , Ma Y , Wibbelsman C , et al. Rate of and risks for regression of cervical intraepithelial neoplasia 2 in adolescents and young women. Obstet Gynecol. 2010;116:1373‐1380. 21099605 10.1097/AOG.0b013e3181fe777f PMC3057366

[29] Deacon JM , Evans CD , Yule R , et al. Sexual behaviour and smoking as determinants of cervical HPV infection and of CIN3 among those infected: a case‐control study nested within the Manchester cohort. Br J Cancer. 2000;83:1565‐1572. 11076670 10.1054/bjoc.2000.1523 PMC2363425

[30] Lycke KD , Kahlert J , Petersen LK , et al. Untreated cervical intraepithelial neoplasia grade 2 and subsequent risk of cervical cancer: population based cohort study. BMJ. 2023;383:e075925. 38030154 10.1136/bmj-2023-075925 PMC10685285

[31] Louvanto K , Aro K , Nedjai B , et al. Methylation in predicting progression of untreated high‐grade cervical intraepithelial neoplasia. Clin Infect Dis. 2020;70:2582‐2590. 31344234 10.1093/cid/ciz677 PMC7286376

[32] Bruinsma F , Lumley J , Tan J , Quinn M . Precancerous changes in the cervix and risk of subsequent preterm birth. BJOG. 2007;114:70‐80. 17083653 10.1111/j.1471-0528.2006.01107.x

