# Supplementary material for: Predictors for regression and progression of actively surveilled cervical intraepithelial neoplasia grade 2: A prospective cohort study
Source: Acta Obstet Gynecol Scand. 2025 Feb 10;104(4):763–73. doi: 10.1111/aogs.15032 (PMC11919748; doi:10.1111/aogs.15032)
Supplement: Supplementary file 1 — Appendix S1. [file AOGS-104-763-s001.docx]

**Appendix S1**

**Cytological criteria for colposcopy referral and timing of colposcopy (Finnish current care guidelines 2023)**

1. Repeated low grade squamous intraepithelial lesion (LSIL) findings on cytology in women under 30 years and all repeated atypical squamous cells of undetermined significance (ASC-US) findings require colposcopy within six months of referral.
2. High grade cytology findings (atypical squamous cells, cannot exclude high grade squamous intraepithelial lesion (HSIL) (ASC-H), HSIL, atypical glandular cells favor neoplasia (AGC-FN) require colposcopy within one month of referral.
3. Atypical glandular cells not otherwise specified (AGC-NOS) require colposcopy within two months of referral or by cytologist’s recommendation.

Working group set up by the Finnish Medical Society Duodecim and the Finnish Colposcopy Association. Cytological Changes in the Cervix, Vagina and Vulva. Current Care Guidelines. The Finnish Medical Society Duodecim. Available online at:<https://kaypahoito.fi>, updated 2023 (Accessed October 17, 2023)

**Low-risk human papillomavirus (HPV) genotypes**

HPV6, 11, 30, 40, 42, 43, 53, 61, 67, 69, 70, 73, 74, 81, 83, 86, 87, 89, 90, and 91

**TABLE S1** The distribution of different HPV genotypes in 243 women with active surveillance of HSIL/CIN2 with and without hierarchial system. Hierarchial system defined as all women having HPV16 were categorized as HPV16 positive group irrespective of other possible HPV genotypes detected.

| High-risk HPV genotypes | |  |  |  |  |
| --- | --- | --- | --- | --- | --- |
| Without  hierarchy N (%) | | With hierarchy | N (%) |  |  |
| hrHPV | 185 (82.6) | hrHPV | 185 (82.6) |  |  |
| HPV16 | 94 (42.0) | HPV16 | 94 (42.0) |  |  |
| other hrHPV^a^ | 121 (54.0) | other hrHPV^a^ | 91 (40.6) |  |  |
| HP18 | 14 (6.3) | HP18 | 10 (4.5) |  |  |
| HPV31 | 31 (13.8) | HPV31 | 29 (12.9) |  |  |
| HPV33 | 14 (6.3) | HPV33 | 12 (5.4) |  |  |
| HPV35 | 8 (3.6) | HPV35 | 7 (3.1) |  |  |
| HPV39 | 7 (3.1) | HPV39 | 3 (1.3) |  |  |
| HPV45 | 9 (4.0) | HPV45 | 5 (2.2) |  |  |
| HPV51 | 12 (5.4) | HPV51 | 8 (3.6) |  |  |
| HPV52 | 23 (10.3) | HPV52 | 16 (7.1) |  |  |
| HPV56 | 6 (2.7) | HPV56 | 4 (1.8) |  |  |
| HPV58 | 10 (4.5) | HPV58 | 8 (3.6) |  |  |
| HPV59 | 6 (2.7) | HPV59 | 5 (2.2) |  |  |
| HPV66 | 10 (4.5) | HPV66 | 8 (3.6) |  |  |
| HPV68 | 10 (4.5) | HPV68 | 5 (2.2) |  |  |
| Low-risk HPV genotypes^b^ | |  |  |  |  |
| Without hierarchy | N (%) | With hierarchy | N (%) | Only lrHPV^b^ genotypes | N (%) |
| lrHPV^b^ | 50 (22.3) | lrhpv^b^ | 36 (16.1) | lrhpv^b^ | 10 (4.5) |
| HPV6 | 4 (1.8) | HPV6 | 3 (1.3) | HPV6 | 2 (0.9) |
| HPV11 | 1 (0.4) | HPV11 | 1 (0.4) | HPV11 | 1 (0.4) |
| HPV30 | 2 (0.9) | HPV30 | 1 (0.4) | HPV30 | 0 (0.0) |
| HPV40 | 0 (0.0) | HPV40 | 0 (0.0) | HPV40 | 0 (0.0) |
| HPV42 | 8 (3.6) | HPV42 | 7 (3.1) | HPV42 | 2 (0.9) |
| HPV43 | 1 (0.4) | HPV43 | 0 (0.0) | HPV43 | 0 (0.0) |
| HPV53 | 6 (2.7) | HPV53 | 4 (1.8) | HPV53 | 0 (0.0) |
| HPV61 | 0 (0.0) | HPV61 | 0 (0.0) | HPV61 | 0 (0.0) |
| HPV67 | 6 (2.7) | HPV67 | 4 (1.8) | HPV67 | 0 (0.0) |
| HPV69 | 0 (0.0) | HPV69 | 0 (0.0) | HPV69 | 0 (0.0) |
| HPV70 | 4 (1.8) | HPV70 | 3 (1.3) | HPV70 | 0 (0.0) |
| HPV73 | 4 (1.8) | HPV73 | 4 (1.8) | HPV73 | 3 (1.3) |
| HPV74 | 5 (2.2) | HPV74 | 4 (1.8) | HPV74 | 2 (0.9) |
| HPV81 | 1 (0.4) | HPV81 | 1 (0.4) | HPV81 | 1 (0.4) |
| HPV83 | 1 (0.4) | HPV83 | 1 (0.4) | HPV83 | 0 (0.0) |
| HPV86 | 1 (0.4) | HPV86 | 1 (0.4) | HPV86 | 0 (0.0) |
| HPV87 | 2 (0.9) | HPV87 | 2 (0.9) | HPV87 | 1 (0.4) |
| HPV89 | 2 (0.9) | HPV89 | 1 (0.4) | HPV89 | 1 (0.4) |
| HPV90 | 8 (3.6) | HPV90 | 5 (2.2) | HPV90 | 1 (0.4) |
| HPV91 | 8 (3.6) | HPV91 | 4 (1.8) | HPV91 | 2 (0.9) |

^a^Other hrHPV genotypes: 18, 31, 33, 35, 39, 45, 51, 52, 56, 58, 59, 66, and 68

^b^Including women only with lrHPV genotypes: 6, 11, 30, 40, 42, 43, 53, 61,67, 69, 70, 73, 74, 81, 83, 86, 87, 89, 90, and 91.

Abbreviations: CIN: cervical intraepithelial neoplasia; HPV: human papillomavirus; hr: high risk; lr: low risk

**TABLE S2** Comparison of the baseline characteristics of 243 women completing or defaulting a 24-month active surveillance of cervical intraepithelial neoplasia 2.

|  | Included in the final analysis |  | Defaulted from the study |
| --- | --- | --- | --- |
|  | N (%) |  | N (%) *P* |
| Age (n=205)  median 26.5, range 17.7-30.9 |  | **Age** (n=38)  median 25.9, range 20.5-30.8 | 0.294 |
| 18-25 years | 89 (43.4) | 18-25 years | 20 (52.6) |
| 26-30 years | 116 (56.6) | 26-30 years | 18 (47.4) |
| Contraception (n=205) |  | **Contraception** (n=36) | 0.793 |
| None | 31 (15.1) | None | 7 (19.4) |
| Condom | 43 (21.0) | Condom | 9 (25.0) |
| COC | 90 (43.9) | COC | 13 (36.1) |
| Other^a^ | 41 (20.0) | Other^a^ | 7 (19.4) |
| Parity (n=202) |  | **Parity** (n=37) | 0.011 |
| 0 | 157 (77.7) | 0 | 23 (62.2) |
| 1 | 33 (16.3) | 1 | 6 (16.2) |
| >1 | 12 (5.9) | >1 | 8 (21.6) |
| Cigarette smoking (n=205) |  | **Cigarette smoking** (n=36) | 0.794 |
| Yes | 92 (44.9) | Yes | 17 (47.2) |
| No | 113 (55.1) | No | 19 (52.8) |
| Referral cytology (n=205) |  | **Referral cytology** (n=38) |  |
| Low-grade cytology^b^ | 47 (22.9) | Low-grade cytology^b^ | 8 (21.1) |
| High-grade cytology^c^ | 154 (75.1) | High-grade cytology^c^ | 26 (68.4)  0.093 |
| NILM | 1 (0.5) | NILM | 0 (0.0) |
| ASC-US | 16 (7.8) | ASC-US | 2 (5.3) |
| LSIL | 30 (14.6) | LSIL | 6 (15.8) |
| ASC-H | 76 (37.1) | ASC-H | 9 (23.7) |
| HSIL | 77 (37.6) | HSIL | 17 (44.7) |
| AGC-NOS | 1 (0.5) | AGC-NOS | 0 (0.0) |
| AGC-FN | 1 (0.5) | AGC-FN | 0 (0.0) |
| NA | 3 (1.5) | NA | 4 (10.5) |
| TZ type (n=205) |  | **TZ type** (n=37) | 0.483 |
| 1 | 150 (73.2) | 1 | 25 (67.6) |
| 2 | 55 (26.8) | 2 | 12 (32.4) |
| Biopsies at baseline (n=202)  median 2, mean 2.5, range 1-4 |  | **Biopsies at baseline** (n=37)  median 2, mean 2.3, range 1-4 | 0.112 |
| 1-2 | 108 (53.5) | 1-2 | 25 (67.6) |
| >2 | 94 (46.5) | >2 | 12 (32.4) |
| ATZ size (n=205) |  | **ATZ size** (n=37) | 0.835 |
| 0-25% | 94 (45.9) | 0-25% | 15 (40.5) |
| 26-50% | 70 (34.1) | 26-50% | 14 (37.8) |
| >50% | 41 (20.0) | >50% | 8 (21.6) |
| RCI score (n=202) |  | **RCI score** (n=37) | 0.731 |
| RCI 0-3 | 77 (38.1) | RCI 0-3 | 13 (35.1) |
| RCI 4-6 | 125 (61.9) | RCI 4-6 | 24 (64.9) |
| HPV at baseline^d^ (n=188) |  | **HPV at baseline**^d^ (n=36) | 0.523 |
| Positive | 162 (86.2) | Positive | 33 (91.7) |
| Negative | 26 (13.8) | Negative | 3 (8.3) |
| hrHPV+^e^ | 155(82.4) | hrHPV+^e^ | 30 (83.3) |
| lrHPV+^f^ | 7 (3.7) | lrHPV+^f^ | 3 (8.3) |
| HPV16+ | 80 (42.6) | HPV16+ | 14 (38.9) |
| other hrHPV+^g^ | 75(39.9) | other hrHPV+^g^ | 16(44.4) |
| HPV18+ | 10 (4.5) | HPV18+ | 0 (0.0) |
| HPV31+ | 22 (11.7) | HPV31+ | 7 (19.4) |
| HPV33+ | 8 (4.3) | HPV33+ | 4 (11.1) |
| HPV35+ | 7 (3.7) | HPV35+ | 0 (0.0) |
| HPV39+ | 3 (1.6) | HPV39+ | 0 (0.0) |
| HPV45+ | 4 (2.1) | HPV45+ | 1 (2.8) |
| HPV51+ | 6 (3.2) | HPV51+ | 2 (5.6) |
| HPV52+ | 12 (6.4.) | HPV52+ | 4 (11.1) |
| HPV56+ | 3 (1.6) | HPV56+ | 1 (2.8) |
| HPV58+ | 8 (4.3) | HPV58+ | 0 (0.0) |
| HPV59+ | 5 (2.7) | HPV59+ | 0 (0.0) |
| HPV66+ | 8 (4.3) | HPV66+ | 0 (0.0) |
| HPV68+ | 5 (2.7) | HPV68+ | 0 (0.0) |

^a^Including progestin pills, hormonal intrauterine device (IUD), progestin implant and copper IUD

^b^Low-grade cytology including ASC-US, LSIL, AGC-NOS

^c^High-grade cytology including ASC-H, HSIL, AGC-FN

^d^Single genotypes presented with all women positive for HPV16 categorized as HPV16+ irrespective of other genotypes detected, for other single genotypes (i.e. HPV18+) cases positive also for HPV16 excluded, multiple infections with other genotypes allowed

^e^hrHPV, high-risk HPV genotypes: 16, 18, 31, 33, 35, 39, 45, 51, 52, 56, 58, 59, 66, and/or 68

^f^lrHPV, low-risk HPV genotypes: 6, 11, 30, 40, 42, 43, 53, 61,67, 69, 70, 73, 74, 81, 83, 86, 87, 89, 90, and/or 91; cases with presence of hrHPV genotypes excluded

^g^Other hrHPV genotypes: 18, 31, 33, 35, 39, 45, 51, 52, 56, 58, 59, 66, and/or 68; i.e. cases with HPV16 excluded

Abbreviations: AGC-FN: atypical glandular cells, favor neoplasia; AGC-NOS: atypical glandular cells not otherwise specified; ASC-H: atypical squamous cells, cannot exclude high grade squamous intraepithelial lesion; ASC-US: atypical squamous cells of undetermined significance; ATZ: atypical transformation zone; COC: combined oral contraceptives; HPV: human papillomavirus; hr: high risk; HSIL: high-grade squamous intraepithelial lesion; NILM: negative for intraepithelial lesion or malignancy; lr: low risk; LSIL: low-grade squamous intraepithelial lesion; TZ: transformation zone; NA: not available; RCI: Reid’s colposcopic index

**TABLE S3** a) Crosstabulation between HPV16 detection and age groups (18-25, 26-30).

|  | HPV16- % (column) | HPV16+ % (column) | Total % (column) |
| --- | --- | --- | --- |
| 18-25 y | N=47 | N=35 | N=82 |
| % (row) | 57.3 % | 42.7 % | 100.0 % |
|  | 43.5 % | 43.8 % | 43.6% |
| 26-30 y | N=61 | N=45 | N=106 |
| % (row) | 57.6 % | 42.5 % | 100.0 % |
|  | 56.5 % | 56.3 % | 56.4% |
| Total  % (row) | N=108  57.4%  100.0 % | N=80  42.6%  100.0 % | N=188  100.0%  100.0% |

b) Outcomes according to HPV16 detection and different age groups during two-year active surveillance of HSIL/CIN2

|  | Complete regression N(%) | Partial regression N(%) | Persistence N(%) | Progression N(%) | Total |
| --- | --- | --- | --- | --- | --- |
| All | 132 (64.4) | 28 (13.7) | 12 (5.9) | 33 (16.1) | 205 |
| HPV16+ and 18-25 y | 22 (62.9) | 3 (8.6) | 3 (8.6) | 7 (20.0) | 35 |
| HPV16+ and 26-30 y | 28 (62.2) | 3 (6.7) | 3 (6.7) | 11 (24.4) | 45 |
| HPV16- and 18-25 y | 28 (59.6) | 13 (27.7) | 3 (6.4) | 3 (6.4) | 47 |
| HPV16- and 26-30 y | 43 (70.5) | 7 (11.5) | 2 (3.3) | 9 (14.8) | 61 |

CIN: cervical intraepithelial neoplasia; HPV: Human papillomavirus; HSIL: high-grade squamous intraepithelial lesion

**TABLE S4** Risk differences and risk ratios for regression and progression of characteristics and baseline findings among 205 women with active surveillance of HSIL/CIN2.

| Regression | | | | Progression | | | |
| --- | --- | --- | --- | --- | --- | --- | --- |
|  | Risk difference  (95% CI) | Risk ratio  (95% CI) | *P* |  | Risk difference  (95% CI) | Risk ratio  (95% CI) | *P* |
| Referral cytology |  |  |  |  |  |  |  |
| non-HSIL^a^  n=87/125 | ref | ref |  | non-HSIL^a^  n=16/125 | ref | ref |  |
| HSIL  n=43/77 | -0.14  (-0.27-0.00) | 0.80  (0.64-1.01) | 0.047 | HSIL  n=17/77 | 0.09  (-0.02-0.20) | 1.72  (0.93-3.21) | 0.083 |
| ATZ size |  |  |  |  |  |  |  |
| ≤25%  n=70/94 | ref | ref |  | ≤25%  n=9/94 | ref | ref |  |
| >25-50%  n=45/70 | -0.10  (-0.24-0.04) | 0.86  (0.70-1.07) | 0.159 | >25-50%  n=12/70 | 0.08  (-0.03-0.18) | 1.79  (0.80-4.01) | 0.151 |
| TZ type |  |  |  |  |  |  |  |
| 1  n=99/150 | ref | ref |  | 1  n=21/150 | ref | ref |  |
| 2  n=33/55 | -0.06  (-0.21-0.09) | 0.91  (0.71-1.16) | 0.427 | 2  n=12/55 | 0.08  (-0.04-0.20) | 1.56  (0.82-2.95) | 0.177 |
| RCI score |  |  |  |  |  |  |  |
| RCI 0-3  n=51/77 | ref | ref |  | RCI 0-3  n=9/77 | ref | ref |  |
| RCI 4-6  n=80/125 | -0.02  (-0.16-0.11) | 0.97  (0.79-1.19) | 0.747 | RCI 4-6  n=24/125 | 0.08  (-0.02-0.17) | 1.64  (0.81-3.35) | 0.161 |
| HPV at baseline |  |  |  |  |  |  |  |
| non-  HPV18 hrHPV^b^  n=89/142 | ref | ref |  | non-  HPV18 hrHPV^b^  n=25/142 | ref | ref |  |
| HPV18+^c^  n=4/10 | -0.23  (-0.54-0.09) | 0.64  (0.30-1.38) | 0.155 | HPV18+^c^  n=1/10 | -0.08  (-0.27-0.12) | 0.57  (0.09-3.77) | 0.537 |
| non-  HPV31 hrHPV^d^  n=79/131 | ref | ref |  | non-  HPV31 hrHPV^d^  n=23/131 | ref | ref |  |
| HPV31+^c^  n=13/22 | -0.01  (-0.23-0.21) | 0.98  (0.67-1.42) | 0.914 | HPV31+^c^  n=5/22 | 0.05  (-0.14-0.24) | 1.29  (0.55-3.04) | 0.562 |
| non-  HPV33 hrHPV^e^  n=91/145 | ref | ref |  | non-  HPV33 hrHPV^e^  n=26/145 | ref | ref |  |
| HPV33+^c^  n=2/8 | -0.38  (-0.69-(-0.07)) | 0.40  (0.12-1.33) | 0.033 | HPV33+^c^  n=2/8 | 0.07  (-0.24-0.38) | 1.39  (0.40-4.86) | 0.615 |

^a^Non-HSIL cytology including negative for intraepithelial lesion or malignancy; atypical squamous cells of undetermined significance; low-grade squamous intraepithelial lesion; atypical glandular cells not otherwise specified; atypical squamous cells, cannot exclude high-grade squamous intraepithelial lesion; atypical glandular cells, favor neoplasia

^b^Non-HPV18 hrHPV including HPV 16, 31, 33, 35, 39, 45, 51, 52, 56, 58, 59, 66, and/or 68

^c^Not including co-infections with HPV16

^d^Non-HPV31 hrHPV including HPV 16, 18, 33, 35, 39, 45, 51, 52, 56, 58, 59, 66, and/or 68

^e^Non-HPV33 hrHPV including HPV 16, 18, 31, 35, 39, 45, 51, 52, 56, 58, 59, 66, and/or 68

Abbreviations: ATZ: atypical transformation zone; HPV: human papillomavirus; hr: high risk; HSIL: high-grade squamous intraepithelial lesions; RCI: Reid’s colposcopic index; TZ: transformation zone

**TABLE S5** Risk ratios of the risk factor combinations for progression of cervical intraepithelial lesion in 205 women with two-year active surveillance

| Progression | | | |
| --- | --- | --- | --- |
|  | Risk difference  (95% CI) | Risk ratio  (95% CI) | *P* |
| High-grade^a^ referral and HPV16+ |  |  |  |
| High grade- HPV16-  n=1/28 | ref | ref |  |
| High grade+ HPV16+  n=17/65 | 0.23  (0.10-0.35) | 7.32  (1.02-52.38) | .012 |
| High grade+ HPV16-  n=11/78 | 0.11  (0.00-0.21) | 3.95  (0.53-29.21) | .131 |
| High grade- HPV16+  n=1/15 | 0.03  (-0.11-0.17) | 1.87  (0.13-27.77) | .646 |
| High-grade^a^ referral and other hrHPV^b^ |  |  |  |
| High grade- other hrHPV-  n=1/23 | ref | ref |  |
| High grade+ other hrHPV+  n=9/54 | 0.12  (-0.01-0.25) | 3.83  (0.51-28.54) | .141 |
| High grade+ other hpHPV-  n=19/89 | 0.17  (0.05-0.29) | 4.91  (0.69-34.79) | .058 |
| High grade- other hrHPV+  n=1/20 | 0.01  (-0.12-0.13) | 1.15  (0.08-17.22) | .919 |
| >50% ATZ and HPV16+ |  |  |  |
| ≤50% ATZ HPV16-  n=9/94 | ref | ref |  |
| >50% ATZ HPV16+  n=8/22 | 0.27  (0.06-0.48) | 3.80  (1.65-8.72) | .001 |
| >50% ATZ HPV16-  n=3/14 | 0.12  (-0.10-0.34) | 2.24  (0.69-7.28) | .188 |
| ≤50% ATZ HPV16+  n=10/58 | 0.08  (-0.04-0.19) | 1.80  (0.78-4.17) | .165 |
| >50% ATZ and high-grade^a^ referral |  |  |  |
| ≤50% ATZ High grade-  n=2/41 | ref | ref |  |
| >50% ATZ High grade+  n=12/33 | 0.31  (0.14-0.49) | 7.45  (1.79-31.00) | .001 |
| >50% ATZ High grade-  n=0/7 | -0.05  (-0.11-0.02) | 0.00  (0.00-0.00) | .551 |
| ≤50% ATZ High grade+  n=19/121 | 0.11  (0.02-0.20) | 3.22  (0.78-13.23) | .075 |
| >50% ATZ and other hrHPV^b^ |  |  |  |
| ≤50% other hrHPV-  n=11/88 | ref | ref |  |
| >50% other hrHPV+  n=2/11 | 0.06  (-0.18-0.29 | 1.45  (0.37-5.72) | .599 |
| >50% other hrHPV-  n=9/25 | 0.24  (0.03-0.44) | 2.88  (1.35-6.16) | .007 |
| ≤50% other hrHPV+  n=8/64 | 0.00  (-0.11-0.11) | 1.00  (0.43-2.34) | 1.000 |

^a^High grade cytology including atypical squamous cells, cannot exclude high-grade squamous intraepithelial lesion (HSIL); HSIL; atypical glandular cells, favor neoplasia

^b^Other hrHPV genotypes: 18, 31, 33, 35, 39, 45, 51, 52, 56, 58, 59, 66, and/or 68; i.e. cases with HPV16 excluded

Abbreviations: ATZ: atypical transformation zone; HPV: human papillomavirus; hr: high risk; HSIL: high-grade squamous intraepithelial lesion
